# Effects of conversion of native cerrado vegetation to pasture on soil hydro-physical properties, evapotranspiration and streamflow on the Amazonian agricultural frontier

**DOI:** 10.1371/journal.pone.0179414

**Published:** 2017-06-13

**Authors:** Rodolfo L. B. Nóbrega, Alphonce C. Guzha, Gilmar N. Torres, Kristof Kovacs, Gabriele Lamparter, Ricardo S. S. Amorim, Eduardo Couto, Gerhard Gerold

**Affiliations:** 1 Department of Physical Geography, Faculty of Geosciences and Geography, University of Goettingen, Goettingen, Germany; 2 Department of Soil and Agricultural Engineering, Federal University of Mato Grosso, Cuiabá, MT, Brazil; Oregon State University, UNITED STATES

## Abstract

Understanding the impacts of land-use change on landscape-hydrological dynamics is one of the main challenges in the Northern Brazilian Cerrado biome, where the Amazon agricultural frontier is located. Motivated by the gap in literature assessing these impacts, we characterized the soil hydro-physical properties and quantified surface water fluxes from catchments under contrasting land-use in this region. We used data from field measurements in two headwater micro-catchments with similar physical characteristics and different land use, i.e. cerrado sensu stricto vegetation and pasture for extensive cattle ranching. We determined hydraulic and physical properties of the soils, applied ground-based remote sensing techniques to estimate evapotranspiration, and monitored streamflow from October 2012 to September 2014. Our results show significant differences in soil hydro-physical properties between the catchments, with greater bulk density and smaller total porosity in the pasture catchment. We found that evapotranspiration is smaller in the pasture (639 ± 31% mm yr^-1^) than in the cerrado catchment (1,004 ± 24% mm yr^-1^), and that streamflow from the pasture catchment is greater with runoff coefficients of 0.40 for the pasture and 0.27 for the cerrado catchment. Overall, our results confirm that conversion of cerrado vegetation to pasture causes soil hydro-physical properties deterioration, reduction in evapotranspiration reduction, and increased streamflow.

## Introduction

Despite accounting for nearly half of all tropical forests and approximately 6% of the Earth’s land surface, tropical dry forests are underrepresented in the literature on tropical forest research [1–3]. Further, tropical dry forests are recognized as one of the world’s most endangered terrestrial ecosystems, as they are threatened by deforestation and climate change impacts [4].

Available empirical data for tropical forests are insufficient for adequate parameterization of water balance models, including the understanding of the effects of deforestation on evapotranspiration and runoff ratios. Therefore, increased efforts with focus on field-based characterizations and catchment processes are recommended to quantify human influence on all aspects of tropical hydrology [5]. Farrick and Branfireun [3] supported this recommendation, adding that standard hydrological metrics such as runoff coefficients also lack comprehensive characterization in tropical dry forests.

The Cerrado ecosystem, commonly called the Brazilian savanna, is South America’s largest tropical dry forest and second-most extensive biome. Although public interest in deforestation in Brazil focuses on the Amazon biome, most of the deforestation has occurred in areas adjacent to the Cerrado-Amazon transition zone [6], also known as the Amazonian agricultural frontier. Approximately 50% of the original 2 million km^2^ of the Cerrado area is under agricultural use [7–9], compromising ca. 80% of the primary cerrado vegetation [10]. Other studies indicate that the conversion of cerrado vegetation will continue to be a dominant process of land-use change in Brazil [11,12].

It is widely known that the removal of forest cover associated with agricultural expansion shifts water balances by reducing evapotranspiration and increasing streamflow [13–15]. Studies evaluating the impacts of land-use change on hydrological processes in the Amazon are relatively common [16–21]. However, assessments of the environmental impacts of the Cerrado conversion into agro-pastoral landscapes are scarce [22–24] despite the importance of the cerrado in provisioning and maintaining ecosystem services such as adequate water quantity and quality [25–27]. Although studies show that land-cover change in the Brazilian Cerrado alters the water balance, e.g. by increasing streamflow [28,29], they do not allow generalizations since they are based mostly on low-resolution datasets. In this biome, water balance components such as streamflow and infiltration, and soil physical properties are poorly understood, especially at field scale in the Cerrado [24,30]. Furthermore, the scarcity of hydrometeorological data and the lack of information on vegetation and geological characteristics are major limitations for a reliable quantification of these land-use change effects.

In fact, most of hydrological characterizations of the Cerrado are often limited to either grey or non-peer reviewed literature, which is difficult to access. Evapotranspiration has been the water balance component studied in greater detail in this biome [31,32]. In more recent studies, the emphasis has been on the use of remote sensing techniques to establish a better understanding of evapotranspiration in large areas of the Brazilian Cerrado [33–38]. However, there are limitations to obtain cloud-free satellite images in this region of Brazil [39], and due to inconsistent field information, studies often have restrictions to apply ground-based validation methods [40].

Burt and McDonnell [41] emphasize that there is a noticeable need for field research to seek new fundamental understanding of catchment hydrology particularly in regions outside of the traditional focus, such as the Cerrado. Due to the lack of data with high temporal and spatial resolution for this region of Brazil, macroscale analyses are often the only alternative. Our study focuses on small headwater catchments because they are the origins of larger rivers, and, as outlined by Guzha et al. [42], hydrological signatures exhibited in these catchments can provide useful indicators of environmental changes in larger areas. Studies using small watersheds in the Brazilian Cerrado are usually more feasible than macro-scale approaches to detected hydrological responses to human impacts regarding land-use and land-cover changes [37,43].

Our hypothesis is that conversion of undisturbed cerrado to pasture leads to soil hydro-physical degradation, increased stream discharge, and reduced evapotranspiration fluxes. In this respect, our study aims to aid filling the gap in the understanding of soil degradation and hydrological processes in active deforestation zones on the Amazonian agricultural frontier in Brazil. The specific objectives were to: i) determine soil hydro-physical properties, and; ii) quantify streamflow and evapotranspiration from two adjacent catchments, whose major difference is the land use (undisturbed cerrado vs. pasture).

## Methods

### Ethics statement

No specific permits were required for our field studies. The accessed areas were privately owned and the respective landowners approved our access during the study period. There was no activity involving sampling or analysis of protected species in our study.

### Study area description

**Fig 1:**
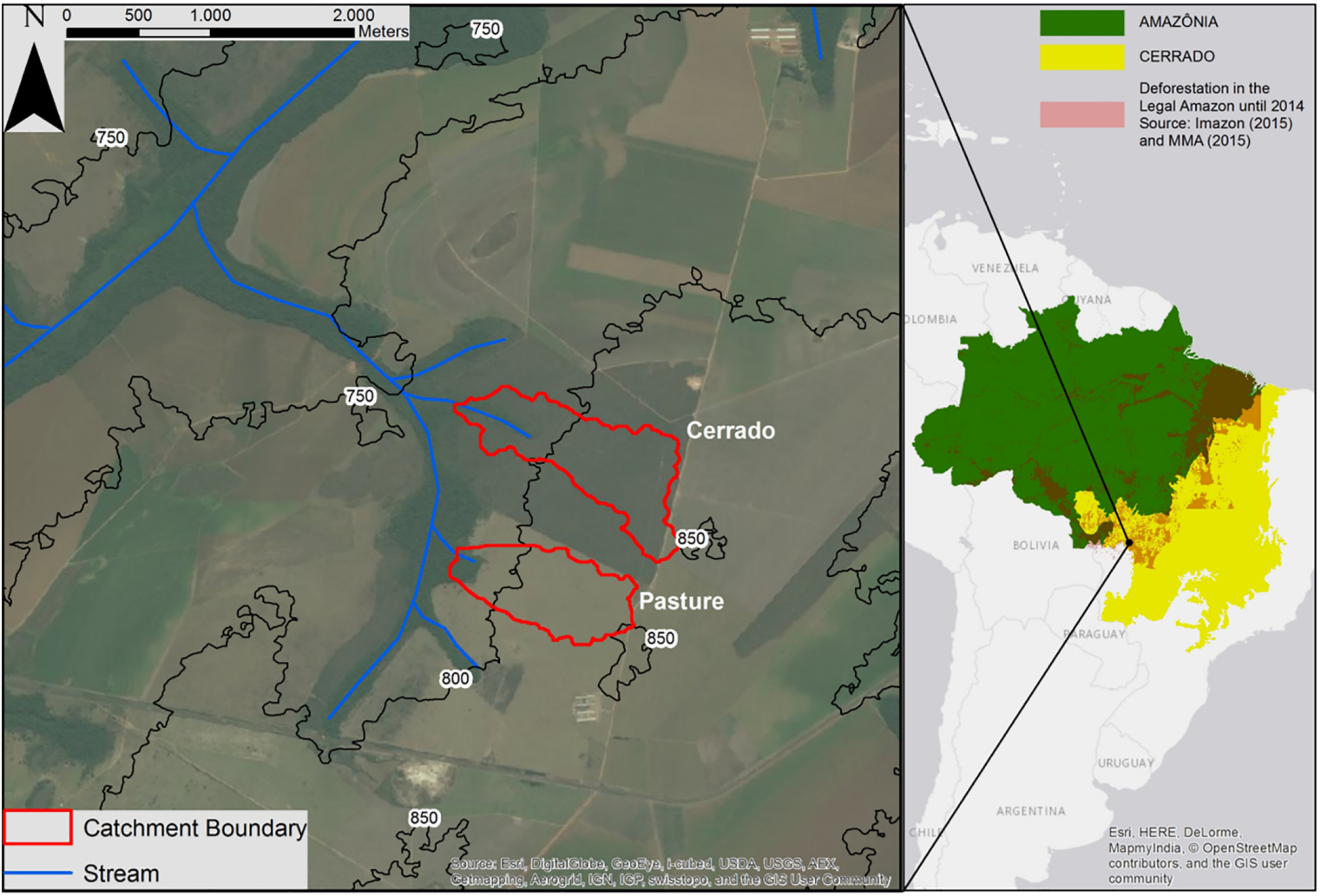
Overview of the Amazon and Cerrado biomes, the deforestation extension in the Legal Amazon, and the location of the cerrado and pasture catchments. Deforestation data from: IMAZON [Internet]; 2016. Available from: http://www.imazongeo.org.br/doc/downloads.php; and MMA [Internet]; 2016. Available from: http://mapas.mma.gov.br/i3geo/datadownload.htm.

We conducted this study in the municipality of Campo Verde (Mato Grosso state, Brazil), situated in the *das Mortes* River basin and in the Cerrado biome (Fig 1). This area is underlain by a Cretaceous sandstone [44]. The soils in this biome are generally highly weathered and acidic with high aluminum concentrations, thus requiring fertilizers and lime for crop production and livestock farming [45]. The climate in this region is tropical wet and dry, and the mean annual precipitation is 1,800 mm yr^-1^; the wet season extends from October to April, and the dry season extends from May to September [46].

We compared two adjacent headwater micro-catchments selected on the basis of their Predominant Land Use (PLU), i.e. cerrado vegetation and pasture for extensive cattle ranching, and monitored them from October 2012 to September 2014. The selected catchments are less than 1 km^2^ in spatial extent, with similar slopes, aspects, soils, and climate. We used the space for time substitution approach for the comparison between the catchments, which it is often used in hydrology to compare adjacent small catchments with similar characteristics and different land cover [47–51]. This method has yielded significant insights in the hydrologic response of landscapes in the absence of historical data and one major different pattern [52].

With an area of 78 ha, the cerrado catchment is located within the boundaries of the *Rancho do Sol* farm (15.797° S, 55.332° W) and is mostly covered by cerrado sensu stricto vegetation. The cerrado sensu stricto is described as a deep-rooting and dense orchard-like vegetation consisting of many species of grasses and sedges mixed with a great diversity of forbs, such as *Leguminosae*, *Compositae*, *Myrtaceae*, and *Rubiaceae* plant species, and trees with an average height of 6 m [45,53–56]. The adjacent pasture catchment (58 ha) is located on the *Gianetta* farm (15.805°S, 55.336°W). In 1993 the original cerrado vegetation in this catchment was removed and replaced by *Brachiaria* grass species for intensive cattle farming. The soils in both micro-catchments are Arenosols (IUSS Working Group WRB, [57]) characterized by a sandy loam texture, and are correlated with *Entisols Quartzipsamments* (Soil Survey Staff, [58]) and *Neossolos Quartzenicos* (Brazilian Soil Classification, [59]).

Although each catchment was selected on the basis of the PLU, gallery forests exist in both micro-catchments following the stream channel. The width of the gallery forest within each catchment varies from 50 to 200 m. The gallery forests have a higher plant diversity compared to the dominant cerrado vegetation [60,61], and they are common formations in the riparian zones in the Cerrado, which occupy about 5% of the Cerrado biome area [62].

### Catchment instrumentation, characterization, and analysis

#### Topographic survey

To define the catchment boundaries and topographic features for the pasture catchment, we used the Quarryman^®^ Auto-Scanning Laser System (ALS) LaserAce Scanner 300p laser profiling system (Measurement Devices Ltd., UK). Due to interferences of the cerrado vegetation in the laser scanner results, we surveyed the cerrado catchment by using a ProMark^™^ differential Global Positioning System (dGPS) instrument (Ashtech, USA). For the survey of the gallery forests, we used the dGPS instrument and a Geodetic Rover System (GRS1) GPS (Topcon, USA) with an integrated TruPulse^®^ 360° B distance measurement system (Laser Technology Inc., USA). We used the topographic data to develop a Digital Elevation Model (DEM) at 5 m resolution for each catchment. Catchment slope distributions and Compound Topographic Index (CTI) were derived from the DEMs. The CTI is a hydrologically-based compound topographic attribute, represented by a steady state wetness index as a function of both the slope and the upstream contributing area [63]. High CTI is represented by areas with greater contributing areas and low slopes. The CTI was computed using the algorithm described by Gessler et al. [64], which was implemented in ArcGIS^®^ by Evans et al. [65].

#### Soil geostatistical analysis and sampling

We delineated transects for soil sampling based on the surface elevation and geostatistical analysis of the clay content to regionalize the soil properties [66–68]. For the surface elevation analysis, we used the DEMs derived from the topographic survey, and for the clay content we collected and analyzed 45 disturbed soil samples at the depth intervals of 0–20 and 40–60 cm from randomly selected points throughout each catchment. We interpolated the clay content results at each soil depth using isotropic variogram analyses and the ordinary kriging method. The variogram results of soil properties as a prerequisite to kriging allow the quantification of the semivariance for any given distance [69].

For the transect delineation only the interpolation of the clay content at 0–20 cm soil depth was used because it showed variogram correlations of 0.94 for the cerrado catchment and 0.83 for the pasture catchment, which were higher than the correlations obtained with the 40–60 cm soil depth. We validated the interpolation results by using the leave-one-out cross-validation method [70], which was based on leaving actual data out one at time and estimating the properties of the location from the neighboring data. We then categorized the surface elevation in 5 equal intervals and clay content in quintiles, and delineated transects from the catchments crest to the stream valley passing over all elevation and clay content categories. We established 15 approximately equally-spaced points along the transects in each catchment to collect in each point one disturbed sample and two undisturbed soil core samples (4.8 cm in diameter and 5.2 cm in height) at depth intervals of 0–10, 10–20, 20–40, and 40–60 cm.

#### Soil physical and hydraulic properties

The disturbed soil samples were analyzed to obtain the particle size distribution, and the undisturbed samples were used to determine bulk density, saturated hydraulic conductivity (K_sat_), particle size distribution, total porosity, macroporosity, microporosity, and field capacity. Particle size distributions of the soils were obtained by using the pipette method [71] after chemical dispersion and removal of organic matter and carbonates. Soil bulk density was estimated by weighing the samples after drying them in an oven at 105°C [72]. K_sat_ was determined by using the constant-head permeameter method. Total porosity was quantified with the cylinder volume method [73]; the macroporosity (pore diameter ≥ 0.05 mm) was determined using the tension table method [73]; and the microporosity was obtained by the difference between the total porosity and the macroporosity. Field capacity moisture content was estimated with the pressure membrane method at -0.01 MPa [74].

#### Rainfall and evapotranspiration

To account for rainfall spatial variability, three tipping bucket rain gauges (0.2 mm resolution) with data loggers (Tinytag^®^, Gemini, UK) were installed in each catchment to record rainfall at 10-min intervals. A WS-GP1 weather station (Delta-T, UK) installed at a farm approximately 7 km from the two catchments (15.741435°S, 55.363134°W) provided total solar radiation, net solar radiation, temperature, relative humidity, wind speed and direction, and rainfall data at 10-min intervals. Using this weather data we quantified the reference evapotranspiration (E_To_) using the standardized reference evapotranspiration equation [75]:

$$E_{To}=\frac{0.408\Delta (R_{n}-G)+\gamma \frac{C_{n}}{T+273}u_{2}(e_{s}-e_{a})}{\Delta +\gamma (1+{C_{d}u}_{2})},$$ (1)

where E_To_ is in mm day^-1^ or mm h^-1^ for daily or hourly time steps), R_n_ is the surface net radiation (MJ m^-2^ day^-1^ or MJ m^-2^ h^-1^ for daily or hourly time steps), G is the soil heat flux density (MJ m^-2^ day^-1^ or MJ m^-2^ h^-1^ for daily or hourly time steps), T is the mean daily air temperature (°C) and u_2_ is the wind speed (m s^-1^) at 2 m height, e_s_ and e_a_ are, respectively, the saturation and actual vapor pressure (kPa), e_s_ − e_a_ is the saturation vapor pressure deficit (kPa), Δ is the slope of vapor pressure curve (kPa °C^-1^), γ is the psychrometric constant (kPa °C^-1^), C_n_ and C_d_ are, respectively, the numerator and denominator constants for the reference type and calculation time step given by ASCE-EWRI [75].

We applied satellite-based image-processing models to improve our E_T_ estimation for the study area. We estimated the evapotranspiration (E_T_) by using a combination of the Surface Energy Balance Algorithm for Land (SEBAL) and Mapping EvapoTranspiration at high Resolution with Internalized Calibration (METRIC™) models, as described by Allen et al. [76]. Both models are based on the energy balance at the land surface. SEBAL is based on latent heat flux as a residual of the energy balance equation, and its principles and computational basis are described in Bastiaanssen et al. [77] and Bastiaanssen [78]. METRIC considers soil and vegetation as a sole source in the estimation of E_T_, and its principles and application procedures are described in Allen et al. [79]. The application of SEBAL has shown to be adequate to quantify the energy balance for the E_T_ estimation for Cerrado landscapes [40,80], and the use of the METRIC model allows to directly integrate a variety of factors, such as orchard architecture, land-use practices, water stress occurrence, and changes in the weather conditions during the day [81,82].

SEBAL was applied by using a composite of spectral bands 1–7 (path 226 and row 071) of all 13 valid satellite scenes from the Landsat 7 Enhanced Thematic Mapper Plus (ETM+) for our study area and period to determine the energy consumed by the E_T_ process; this is calculated as a residual of the surface energy equation (Eq (2)) using the software ERDAS Imagine^®^ v. 14 (Hexagon AB, USA). To match the satellite spatial extension, we used a 90-m-resolution DEM (Shuttle Radar Topography Mission, version 4.1, [83]) cropped to the study area to adjust the surface temperature according to the differences in elevation and to derive surface slope and aspect information as required in SEBAL to estimate solar radiation [79]. The Earth-Sun distance parameter, also required by SEBAL, was obtained from Chander et al. [84] when not available in the satellite metadata file.

$$LE=R_{n}-G-H,$$ (2)

where LE is the latent heat flux, R_n_ is the instantaneous net radiation, G is the soil heat flux, and H is the sensible heat flux (all in W m^-2^).

METRIC was used to compute the instantaneous E_T_ from the obtained latent heat flux from SEBAL for each pixel within the catchments at the instant of satellite overpass (Eq (3)). We used two anchor points to define the limit conditions by means of a cold pixel (15.7402° S, 55.5292° W) and a hot pixel (15.7264° S, 55.3325° W) for the energy balance over the study area for the internal calibration of sensible heat flux of METRIC [79].

$$E_{Tinst}=3600\frac{LE}{\lambda \rho_{w}},$$ (3)

where E_Tinst_ is the instantaneous E_T_ (mm h^-1^), 3600 is the time conversion from seconds to hours, *ρ*_w_ is the density of water (~ 1000 kg m^-3^), and *λ* is the latent heat of vaporization (J kg^-1^) representing the heat absorbed when one kg of water evaporates and it is computed as:

$$\lambda =[2.501-0.00236(T_{s}-273.15)]\times 10^{6},$$ (4)

where T_s_ is the surface temperature (K).

We applied the evaporative fraction (E_TrF_) and daily E_To_ to estimate the actual daily E_T_ assuming that the E_TrF_ is constant during a day [79] according to Eq (5). Additionally, the Penman–Monteith equation, which we used to estimate E_To_, is known to well-represent the impacts of advection [76]. The E_T_ values for each type of land use were area-weighted and summed to obtain the total actual evapotranspiration estimation for each catchment.

$$E_{T}=E_{TrF}E_{To}.$$ (5)

The E_TrF_ is calculated as the ratio of the E_Tinst_ derived for each pixel to the E_To_ at an hourly time step computed from weather data at the time of the satellite overpass [76,85] using Eq (6). To quantify the E_T_ we used the mean and the respective ±1 standard deviation of the obtained values for E_TrF_ for the wet and dry seasons, separately, considering all valid pixels within each catchment domain. Table 1 shows the description of the satellite scenes, the main local weather data at the satellite overpass time, and the respective E_TrF_ values for the study areas. Some results were not available due to cloud masking or Scan Line Corrector-Off malfunction [86].

$$E_{TrF}=\frac{{ET}_{inst}}{E_{To}}.$$ (6)

**Table 1:** Satellite scenes description, weather data at the satellite overpass time, and E_TrF_ values.

Landsat 7 ETM+ scene description				Weather station				ETrF			
Date	Satellite overpass time (GMT)	Relative Earth-Sun distance^a^	Solar zenith angle cosine^b^	Air temperature (°C)	Relative humidity (%)	Wind speed (m s^-1^)	Surface net radiation (MJ m^-2^ h^-1^)	Cerrado		Pasture	
								GF	PLU	GF	PLU
09 Oct 12	13:41	0.99861	0.882	29.5	49%	3.2	612	1.09	0.93	1.25	0.72
02 Mar 13	13:41	0.99108	0.832	26.2	75%	4.6	532	1.21	0.92	1.07	0.64
08 Jul 13	13:41	1.01668	0.652	29.0	34%	2.8	648	0.63	0.52	0.66	0.16
10 Sep 13	13:41	1.00698	0.811	30.9	30%	5.3	558	0.61	0.37	0.70	0.19
26 Sep 13	13:41	1.00250	0.855	27.7	28%	1.9	601	0.84	0.52	0.77	0.15
13 Nov 13	13:41	0.98961	0.905	27.0	66%	3.4	672	1.10	0.76	1.17	N/A^c^
29 Nov 13	13:41	0.98641	0.896	27.9	68%	2.1	667	N/A^c^	1.29	N/A^c^	0.97
01 Feb 14	13:42	0.98536	0.847	27.0	69%	2.9	495	N/A^c^	1.19	N/A^c^	0.51
06 Apr 14	13:42	1.00069	0.791	27.4	73%	2.1	630	1.14	0.96	0.94	0.60
25 Jun 14	13:43	1.01647	0.651	24.5	67%	2.1	430	1.20	0.98	0.96	0.47
11 Jul 14	13:43	1.01661	0.659	20.9	80%	3.4	453	1.20	0.96	1.10	0.45
12 Aug 14	13:43	1.01332	0.725	27.3	46%	2.0	510	0.91	0.68	0.77	0.30
13 Set 14	13:43	1.00620	0.823	30.2	38%	1.8	458	1.16	0.89	1.03	0.61

GF = Gallery Forest area, PLU = Predominant Land Use area

^a^ Inverse square and dimensionless.

^b^ Dimensionless.

^c^ Not available due to cloud masking or Scan Line Corrector-Off malfunction.

#### Catchment discharge and hydrograph analysis

At the outlet of each catchment, an adjustable weir was installed. During the wet season the weirs were maintained as rectangular weirs, and during the dry season a v-notch contraction was inserted. At a distance of 2 m upstream of each weir, a DS 5X (OTT, USA) multiparameter probe was installed to measure, among other variables, the water level at 10-min intervals. For the rectangular weir, we used the standard flow equation (Eq (7)) based on the Bernoulli equation to quantify stream discharge. For the v-notch weir, the Kindsvater–Shen equation (Eq (8)) and respective calibration adjustment functions (Eqs (9) and (10)) were used to quantify discharge:

$$Q=\frac{2}{3}C_{dr}b\sqrt{2g}h^{\frac{3}{2}},$$ (7)

$$Q=\frac{8}{15}C_{e}\sqrt{2g}\;\tan\;\left(\frac{\theta }{2}\right)\;{h_{e}}^{\frac{5}{2}},$$ (8)

$$K_{h}=0.001[\theta (1.395\theta -4.296)+4.135],$$ (9)

$$C_{e}=\theta (0.02286\theta -0.05734)+0.6115,$$ (10)

where Q is the discharge over the weir (m^3^ s^-1^), C_dr_ and C_e_ are the effective dimensionless discharge coefficients for the rectangular and v-notch weirs, respectively, b is the weir length (m), θ is the v-notch’s angle (radians), h is the upstream head above the weir’s crest (m), h_e_ is the effective head (h + K_h_), and K_h_ is the head-adjustment factor.

In each catchment, we conducted discharge calibration measurements with an acoustic digital current meter (ADC, OTT, USA) to estimate the C_dr_ factor for each catchment. The obtained C_dr_ values were 0.74 for the cerrado catchment and 0.65 for the pasture catchment. The discharged data were normalized by the correspondent catchment area to allow comparisons between the catchments. To estimate the total streamflow, we used the mean discharge values for each wet and dry seasons. Additionally, we applied ±1 standard deviation of the mean of each wet and dry seasons to the discharge-gap days in order to estimate the total error.

The discharge time series were analyzed with the recursive digital filter method [87] implemented in the Web GIS-based Hydrograph Analysis Tool (WHAT) for baseflow separation [88,89]. The baseflow index (BFI) was computed as the ratio of baseflow to total discharge. The runoff coefficient (R_C_) was determined as the ratio of total discharge to total rainfall. Flow-duration curves (FDCs) were derived from the daily discharge data in order to compare the differences in high, low, and median flows between the catchments [90], and catchment flashiness indices were obtained using the method described by Baker et al. [91].

#### Statistical analyses

Pearson’s correlation analysis was applied to test the relationships between the soil properties, and between the rainfall daily values in each catchment. The results were compared using two sample t-test for the data with normal distribution (soil properties), and a nonparametric test (Mann-Whitney U) in the other cases (rainfall, E_T_, and streamflow), to determine whether the results were significantly different. The significance threshold was set at .05.

## Results

### Catchment physiographic attributes

**Fig 2:**
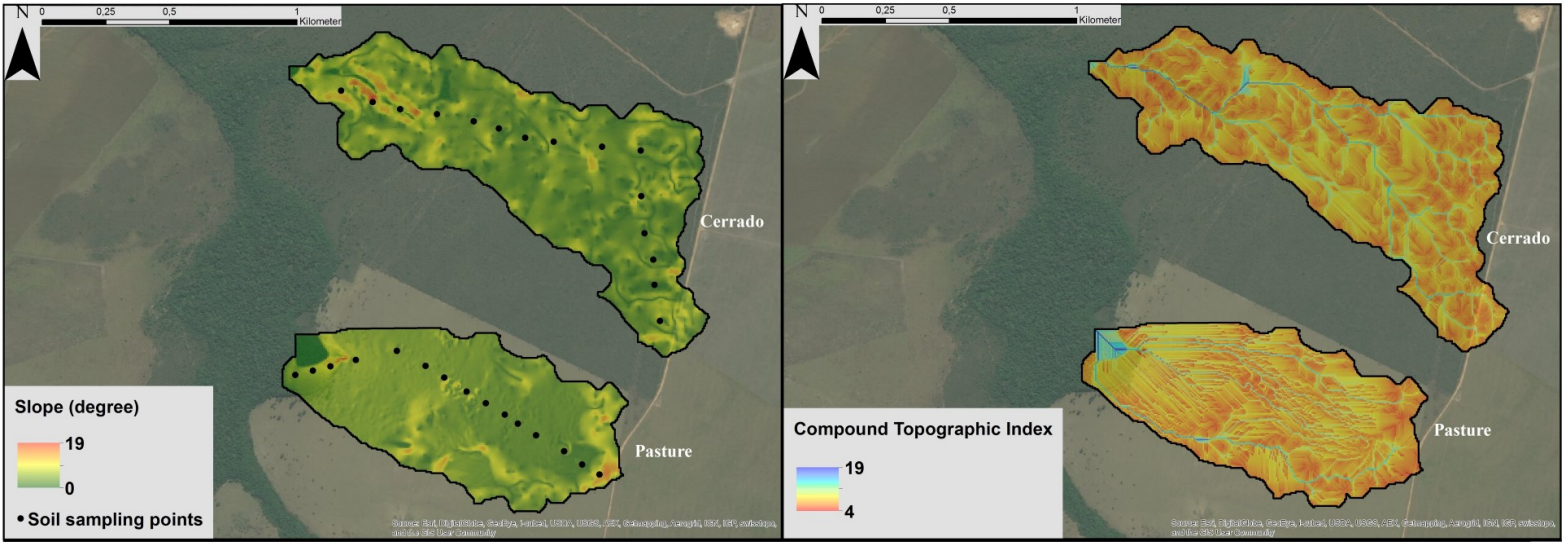
Slope, soil sampling points, and Compound Topographic Index (CTI) in the cerrado and pasture catchments.

The soil sampling points, the slope distribution, and the CTI for each catchment are shown in Fig 2. The cerrado and pasture catchments have similar slope ranges with most of the values between 0 and 10° and an average of approximately 8°. In both catchments over 95% of the area shows CTI values ranging between 5 and 12, and areas with CTI over 10 have linear form extending from the crest to the outlet of the catchments, which indicates the surface flow pathways.

Table 2 shows a summary of the topographic characteristics of the catchments. The data are distinguished for the gallery forest and the PLU areas. The topographic survey shows that the gallery forests cover approximately 7% of the total areas in both catchments.

**Table 2:** Summary of catchments’ physical and topographic characteristics.

	Cerrado catchment			Pasture catchment		
	Gallery Forest	PLU Area	Total Area	Gallery Forest	PLU Area	Total Area
Area (km^2^) (% of total)	0.05 (6.4%)	0.73 (93.6%)	0.78 (100%)	0.04 (6.9%)	0.54 (93.1%)	0.58 (100%)
Predominant land cover	Cerrado sensu stricto vegetation			Grassland (*Brachiaria* species)		
Soil type	Arenosols			Arenosols		
Soil texture	Sandy loam			Sandy loam		
Aspect	E-W			E-W		
Average Elevation (m)	770	814	811	775	821	818
Average slope (°)	7.6	4.6	4.8	3.9	4.4	4.4

### Soil physical and hydraulic properties

Table 3 shows that the cerrado and pasture catchments have comparable soil properties. The pasture catchment shows a greater bulk density (*p* < .0001) at 0–40 cm depth and a lower total porosity (*p* ≈ .0001) at 0–10 cm soil depth compared to the cerrado catchment. Our findings confirm results from Valpassos et al. [92], who reported greater bulk densities in the topsoil of a pasture compared to an area covered by cerrado vegetation. The gallery forest and the PLU areas of the cerrado catchment do not show significant differences in total porosity and bulk densities with identical bulk density results at 0–10 cm soil depth (1.43 ± 9% g cm^-3^), whereas these properties found in the gallery forest area of the pasture catchment are significantly smaller than those in its PLU area (*p* < .0001), especially at 0–20 cm soil depth.

**Table 3:** Summary of the soil properties.

Catchment	Depth interval (cm)	BD (g cm^-3^)	TP (%)	MaP (%)	MiP (%)	FC (%)	K_sat_ (mm h^-1^)	Sand (%)	Silt (%)	Clay (%)
	0–10	1.43 ± 9% (1.43 ± 9%)	49.2 ± 8% (49.4 ± 10%)	31.8 ± 12% (26.9 ± 13%)	17.4 ± 35% (22.5 ± 36%)	15.9 ± 36% (20.5 ± 40%)	559.5 ± 38% (361.1 ± 15%)	85.8 ± 10% (83.7 ± 8%)	2.4 ± 95% (2.64 ± 109%)	11.9 ± 54% (13.6 ± 27%)
Cerrado	10–20	1.47 ± 6% (1.55)	45.8 ± 5% (45.7)	30.8 ± 18% (28.3)	15.0 ± 32% (17.5)	13.2 ± 37% (16.1)	611.7 ± 45% (363.4)	88.9 ± 2% (81.3 ± 9%)	1.5 ±75% (3.73 ± 78%)	9.6 ± 10% (15.0 ± 29%)
	20–40	1.52 ± 4%	42.9 ± 7%	27.0 ± 18%	15.9 ± 32%	14.7 ± 32%	515.56 ± 56%	87.4 ± 1%	1.3 ± 37%	11.3 ± 7%
	40–60	1.51 ± 3%	42.1 ± 2%	25.2 ± 24%	16.9 ± 36%	15.6 ± 36%	509.6 ± 33%	86.2 ± 1%	1.9 ± 49%	11.9 ± 10%
	0–10	1.56 ± 3% (1.23 ± 10%)	44.4 ± 3% (53.5 ± 4%)	28.1 ± 8% (33.0 ± 9%)	16.4 ± 10% (20.4 ± 16%)	15.5 ± 10% (19.3 ± 19%)	399.0 ± 40% (297.3 ± 52%)	88.4 ± 1% (86.0 ± 2%)	1.5 ± 40% (2.1 ± 8%)	10.1 ± 9% (11.9 ± 12%)
Pasture	10–20	1.57 ± 3% (1.37 ± 3%)	45.7 ± 3% (49.8 ± 5%)	32.1 ± 5% (32.0 ± 10%)	13.6 ± 10% (17.8 ± 9%)	12.9 ± 9% (16.6 ± 13%)	655.6 ± 15% (666.5 ± 46%)	89.2 ± 1% (86.6 ± 2%)	0.9 ± 97% (2.1 ± 48%)	9.9 ± 10% (11.3 ± 22%)
	20–40	1.56 ± 3% (1.41 ± 3%)	46.4 ± 4% (50.3 ± 1%)	32.9 ± 7% (33.6 ± 7%)	13.5 ± 10% (16.7 ± 16%)	12.8 ± 10% (15.8 ± 18%)	705.1 ± 17% (611.3 ± 25%)	87.8 ± 1% (86.7 ± 2%)	1.7 ± 28% (1.9 ± 27%)	10.5 ± 5% (11.4 ± 14%)
	40–60	1.52 ± 3% (1.44 ± 4%)	43.0 ± 6% (46.5 ± 11%)	28.8 ± 7% (30.2 ± 12%)	14.3 ± 6% (16.3 ± 10%)	13.4 ± 8% (15.7 ± 11%)	510.4 ± 30% (411.8 ± 24%)	88.6 ± 1% (88.8 ± 2%)	1.3 ± 39% (1.4 ± 67%)	10.1 ± 10% (9.8 ± 6%)

BD = Bulk Density, TP = Total Porosity, MaP = Macroporosity, MiP = Microporosity, FC = Field Capacity.

Results are expressed in terms of average and relative standard deviation. The results between parentheses are exclusively for the gallery forest areas, and the results without parentheses are related to the Predominant Land Use (PLU) areas of each micro-catchment.

**Fig 3:**
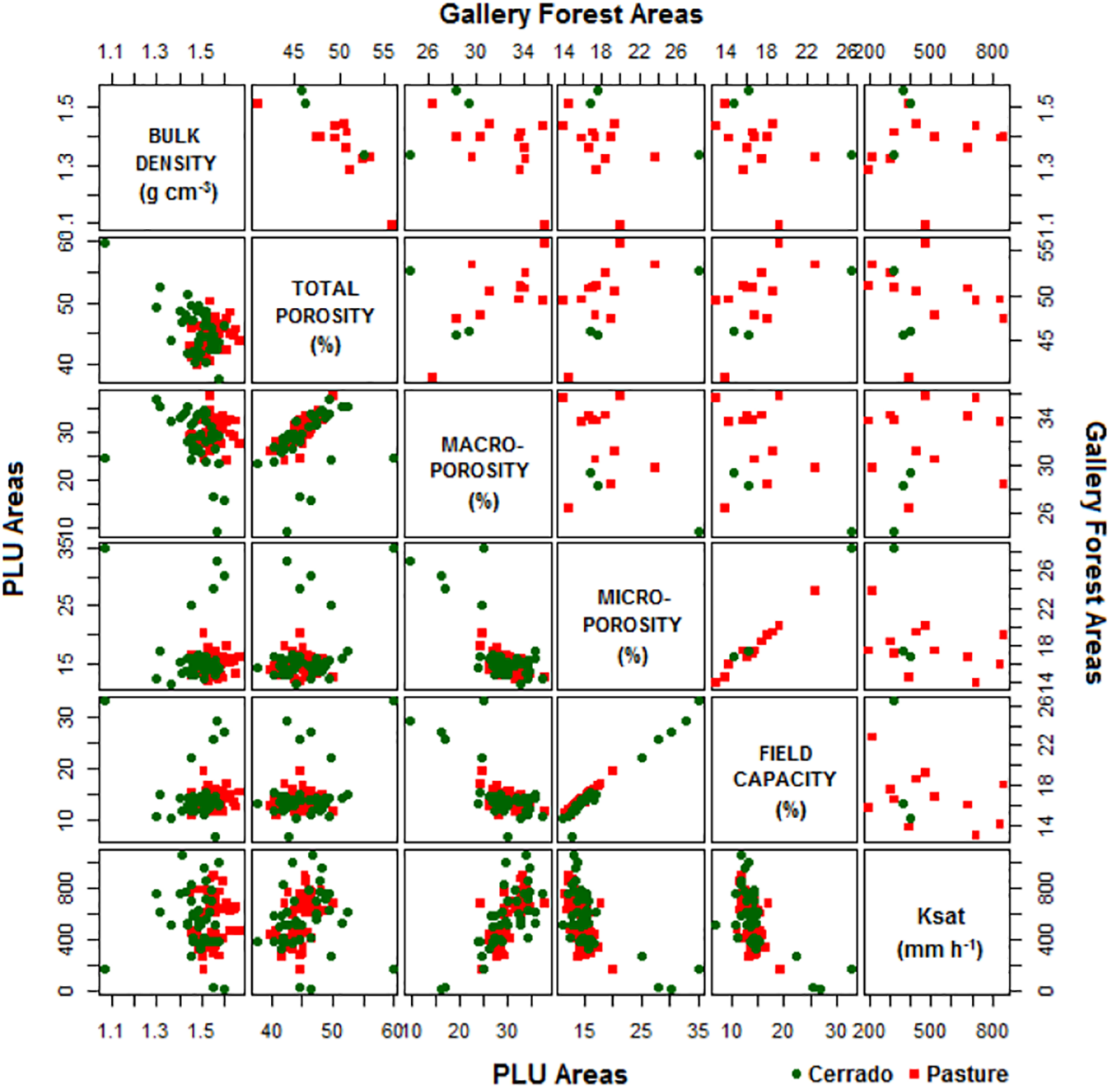
Scatter-plot matrix of soil properties values in the gallery forest (upper panel) and PLU (lower panel) areas in the cerrado and pasture catchments.

Fig 3 shows the relationship between the soil properties in the gallery forest (upper panel) and PLU (lower panel) areas in the cerrado and pasture catchments. As expected, in both catchments the total porosity inversely correlates with the bulk density, and a high correlation (0.98, *p* < .0001) between the microporosity and the field capacity. The microporosity and macroporosity in both catchments exhibited comparable values, with a predominance of the macroporosity between 60 and 70% of the total porosity. In the PLU areas of the cerrado and pasture catchments, there is a positive correlation between the macroporosity and K_sat_ of 0.74 (*p* < .0001) and 0.68 (*p* < .0001), respectively.

**Fig 4:**
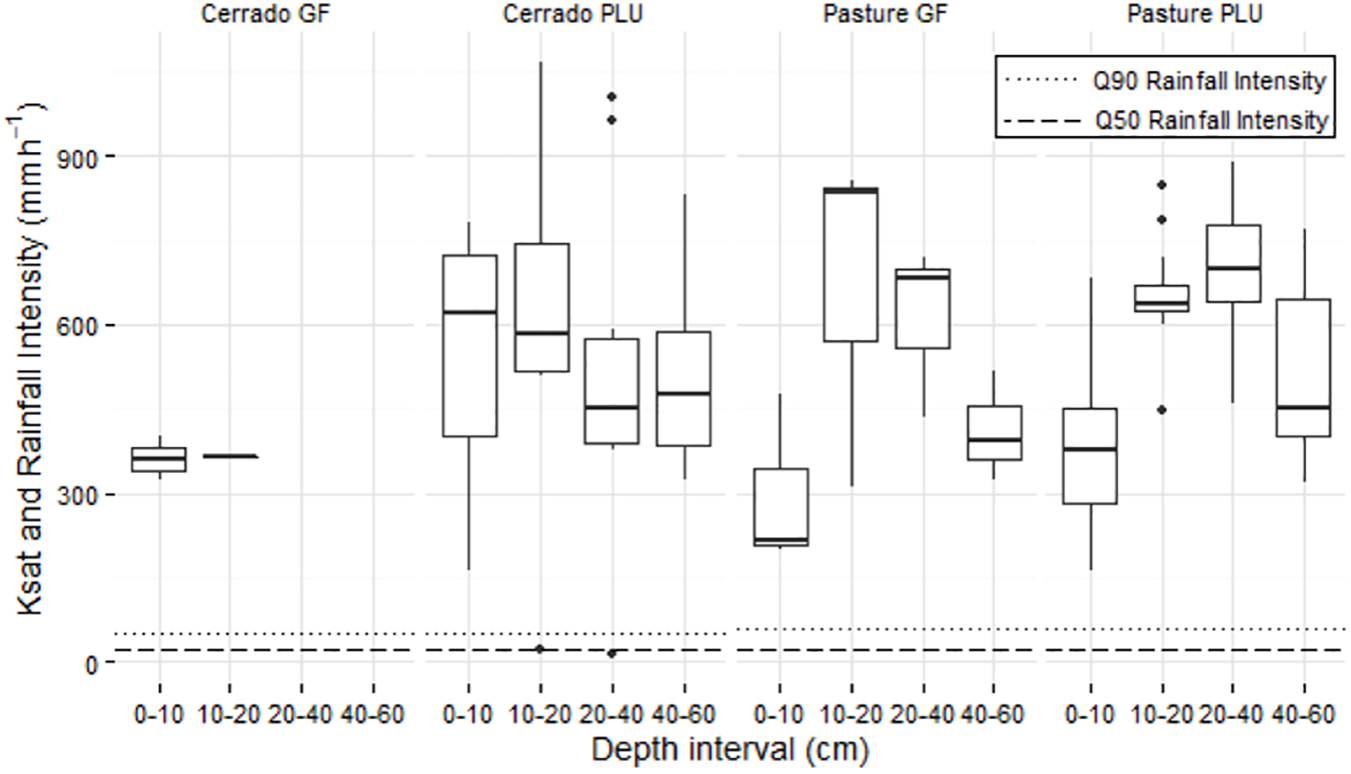
Boxplot of the K_sat_ results, and the 50^th^ and 90^th^ percentiles of the rainfall intensity in the cerrado and pasture catchments.

The K_sat_ distribution for the catchments is shown in Fig 4. The K_sat_ values found in the 0–10 cm soil depth in the PLU areas of the cerrado (559.5 ± 38% mm h^-1^) and pasture (399 ± 40% mm h^-1^) catchments are significantly different (*p* < .05). Martínez and Zink [93] and Zimmerman et al. [94] also found significantly smaller infiltration rates in pasturelands when compared to nearby areas covered by natural forests. In relation to the rainfall intensities in these catchments, the K_sat_ indicate a high infiltration capacity in both catchments, which generally exceeds the rainfall intensities. This is related to the sandy soil texture and the high macroporosity, which is typical for Arenosols. Our results are in accordance with findings of Scheffler et al. [95] who analyzed soil hydraulic properties of catchments with sandy-loam soil texture ca. 450 km from our study area and found K_sat_ values up to 1,200 mm h^-1^.

### Rainfall characteristics

**Fig 5:**
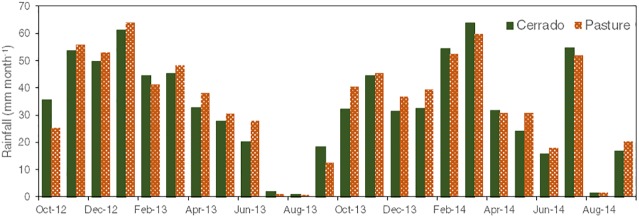
Monthly rainfall per catchment.

The monthly total rainfall in each micro-catchment during the two-year study period is shown in Fig 5. Between October 2012 and September 2014, the total rainfall was 3,392 mm in the cerrado catchment, and 3,560 mm in the pasture catchment. For both catchments, the wet season in 2013–2014 had a smaller contribution to the total annual rainfall than in 2012–2013, which was caused by some atypical rainstorms in the dry season of 2014. The greatest daily rainfall values were recorded on March 2, 2014, for the cerrado catchment, and on January 30, 2013, for the pasture catchment, both at 64 mm d^-1^.

The difference between the catchments’ daily rainfall in the study period is not significant, showing a coefficient of determination of 0.93 (*p* < .0001). We also could not find any significant difference in the rainfall intensity patterns between the cerrado and pasture catchments. In both catchments, the majority of the rainstorms occurred between noon and mid-afternoon with a mean intensity of 28 mm h^-1^, peaks intensities up to 130 mm h^-1^, and a duration between 30 and 90 min.

### Evapotranspiration

**Fig 6:**
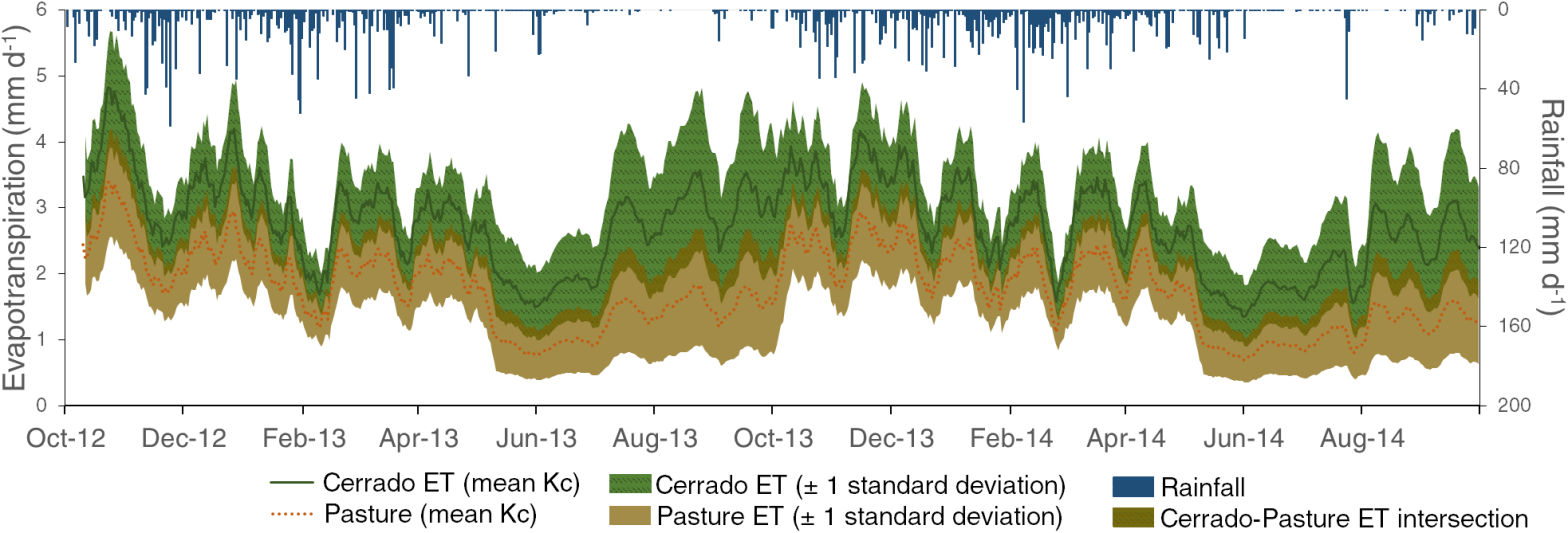
10-day moving average of evapotranspiration, and daily areal average rainfall for the cerrado and pasture catchments.

The daily values of E_T_ are shown in Fig 6. The daily E_T_ was significantly greater in the cerrado catchment (*p* < .0001). In the PLU areas, the average E_T_ was 2.7 mm d^-1^ for the cerrado catchment and 1.7 mm d^-1^ for the pasture catchment. In the gallery forest areas, average daily E_T_ was 3.3 and 2.7 mm d^-1^ for the cerrado and pasture catchments, respectively. The average annual E_T_ was 1,004 ± 24% mm in the cerrado catchment and 639 ± 31% mm pasture catchment. Our results are comparable to E_T_ values for cerrado sensu stricto vegetation ranging between 822 and 1,010 mm yr^-1^ found by Giambelluca et al. [32], Oliveira et al. [37], and Dias et al. [96] who applied eddy-covariance measurements, remoting sensing techniques, and a water balance model, respectively. Da Silva et al. [40] found maximum values between 6 and 7 mm d^-1^ during the wet season for an area covered by cerrado vegetation (mostly sensu stricto type), which are in the same range of the maximum values we found.

Our E_T_ results for the grassland vegetation are in accordance with Dias et al. [96] who used a water balance simulation model and found E_T_ at 567 mm yr^−1^ in the Cerrado-Amazon ecotone, and with Andrade et al. [36] who used remote sensing techniques and found the daily E_T_ varying between 1.5 and 2 mm d^-1^ in the Cerrado biome. In a macro-scale analysis for the Mato Grosso state, Lathuillière et al. [33] reported a range of greater values (822–889 mm yr^-1^) for pasturelands compared to our study; we attribute this difference to the state of degradation of the grassland vegetation in the pasture catchment, which is accredited to reduce the E_T_ [36].

### Streamflow

**Fig 7:**
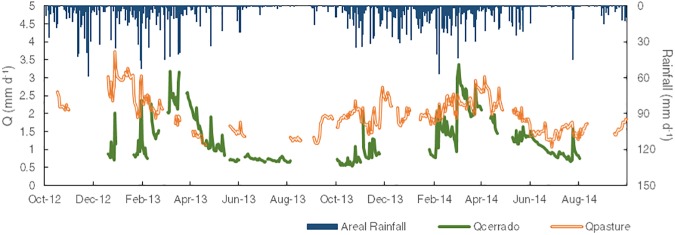
Daily discharges and areal average rainfall for the cerrado and pasture catchments.

The daily discharge values are shown in Fig 7. Due to equipment failure, this time series includes some data gaps. The mean stream discharge was 1.24 mm d^-1^ in the cerrado catchment, and 1.96 mm d^-1^ in the pasture catchment. During the wet season, the mean stream discharge was 1.49 mm d^-1^ in the cerrado catchment, and 2.20 mm d^-1^ in the pasture catchment. In the dry season, the stream discharge was 0.92 mm d^-1^ in the cerrado catchment, and 1.58 mm d^-1^ in the pasture catchment.

Table 4 shows a summary of the hydrological indices derived for the study catchments. During the two-year study period, the daily streamflow was significantly greater (*p* < .0001) in the pasture catchment (1,416 ± 7% mm) compared to the cerrado catchment (914 ± 18% mm). We found R_C_ values of 0.27 for the cerrado and 0.40 for the pasture. Dias et al. (2015) found R_C_ of 0.25 for a cerrado catchment and 0.58 for a pasture catchment using a model based on water balance equations while Tomasella et al. [97] reported a R_C_ of 0.38 for a pasture catchment. The flashiness indices are generally small, particularly for the pasture catchment with indices as low as 0.05. The catchment’s streamflow decreased by 27% from the wet to the dry season while the decrease in the cerrado catchment was 40%.

**Table 4:** Total streamflow and hydrological indices.

	Cerrado		Pasture	
	2012–2013	2013–2014	2012–2013	2013–2014
Mean streamflow (mm yr^-1^)	453	461	724	692
Runoff Coefficient (R_C_)	0.29	0.25	0.45	0.35
Flashiness	0.1145	0.1015	0.0567	0.0517
Baseflow Index (BFI)	0.96	0.97	0.98	0.96

**Fig 8:**
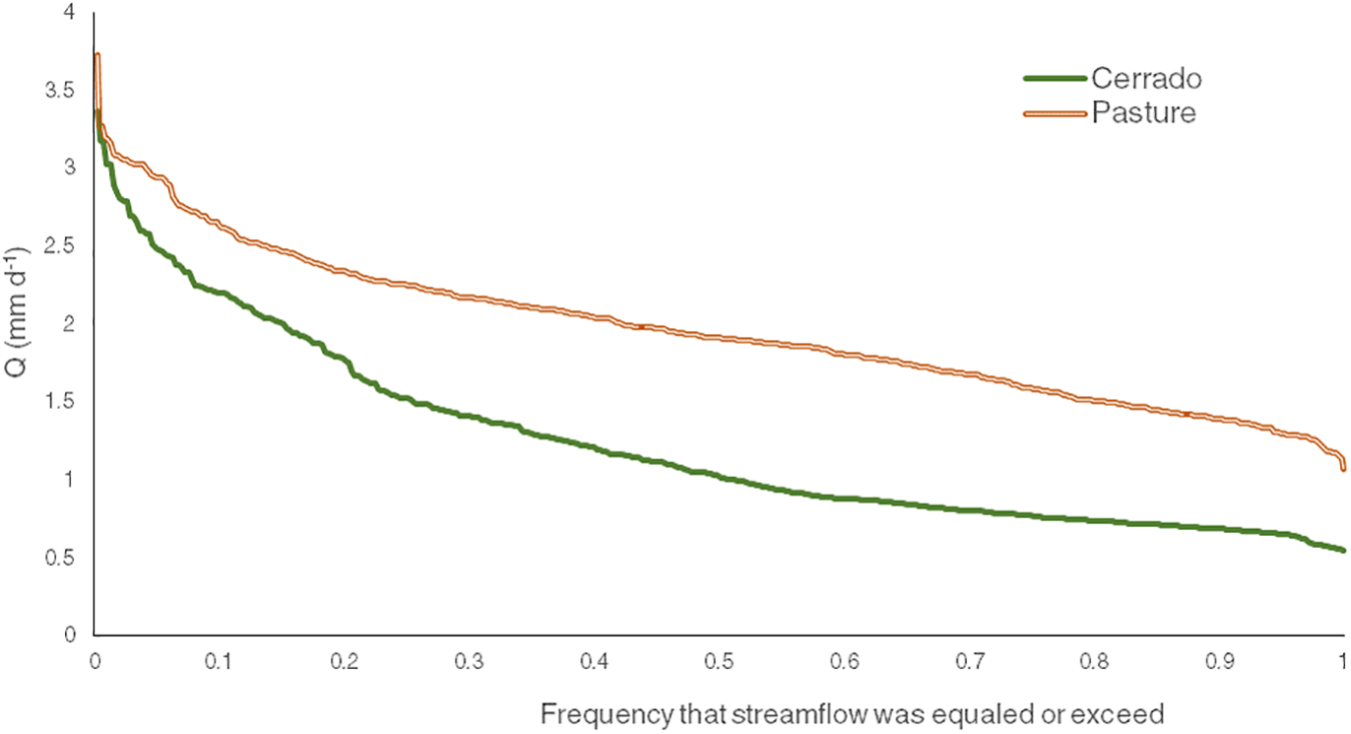
Flow-duration curves of daily discharge for the cerrado and pasture catchments.

The FDCs (Fig 8) of the two catchments show differences in the low flows (Q95) with the cerrado catchment exhibiting the smaller values and greater decrease. Flows with 20% or greater probability of exceedance are higher in the pasture than in the cerrado by an average of 82%. The FDCs curves show a flat slope from the middle to the low flows, supporting that low flows are sustained by the baseflow contribution. This is confirmed by the BFI results, which show a high baseflow contribution to total streamflow in both catchments, with ratios higher than 95%. Total quickflow contribution under 5% was also found in other areas of Cerrado at plot [24] and micro-catchment scales [98–100].

## Discussion

The pasture catchment showed significantly greater bulk densities and smaller K_sat_ and total porosity at the topsoil. Findings like these have been attributed to soil compaction as a consequence of deforestation, cattle grazing and machinery use, e.g. [101–104]. Although we found significantly smaller K_sat_ values in the pasture catchment, these values exceed the observed peak rainfall intensities, which are likely to restrain Hortonian overflow generation and consequently limit the quickflow contribution (< 5%) to the streamflow in both catchments. Zimmerman et al. [94] found similar results in a study on deforested areas in the Amazon basin, showing that the K_sat_ reduction due to land-use change had no significant impact on quickflow generation in those areas. We associate the K_sat_ results to the high macroporosity in both catchments, which has a known effect on soil permeability [105,106]. While macroporosity values around 10% maintain adequate soil permeability [107], our results show a macroporosity of approximately 30% for both catchments. The presence of macroporosity is related to preferential flow [108], which often limits the overflow generation. In fact, our hydrograph analysis shows that baseflow is a major driver of streamflow in both catchments, with BFI over 95%.

Table 5 shows a compilation of the daily and annual E_T_ and Q results for both catchments. The cerrado catchment had the greater E_T_ compared with the pasture catchment. While the mean E_T_ decreased 45% in the pasture catchment from the wet to the dry season, the E_T_ in the cerrado catchment was reduced by 24%. We attribute this result to the canopy cover in the cerrado vegetation with leaf area index values ranging from approximately 0.7 to 1.1 throughout the year [109] and with root lengths sufficient to reach deep soil horizons [56], which ensures E_T_ rates at 2.32 ± 24% mm d^-1^ during the dry season.

**Table 5:** Daily and annual evapotranspiration and streamflow rates.

Catchment	Evapotranspiration			Streamflow		
	Dry (mm d^-1^)	Wet (mm d^-1^)	Annual (mm yr^-1^)	Dry (mm d^-1^)	Wet (mm d^-1^)	Annual (mm yr^-1^)
**Cerrado**	2.32 ± 24%	3.06 ± 26%	1,004 ± 24%	0.92 ± 27%	1.49 ± 46%	457 ± 18%
**Pasture**	1.19 ± 44%	2.15 ± 27%	639 ± 31%	1.58 ± 15%	2.20 ± 20%	708 ± 7%

E_T_ is a major component of the water balance in tropical regions [5]. As reported in other studies [50,110], the differences in E_T_ between native vegetation and grassland plays a major role in the streamflow dynamics. Our results confirms trend analyses and water balance modelling studies at the macro-scale (*das Mortes* River basin), which show an increase of streamflow due to the deforestation of the cerrado vegetation [29,111]. In fact, the conversion of native vegetation to croplands and pasturelands in the Mato Grosso state resulted in a 25% decrease in E_T_ [33], and that water export increases up to fourfold in agricultural areas due to the reduction of E_T_ [112]. Our results are also consistent with those of other studies that reported decreases in E_T_ [37,96] and increases in discharge [26,28,42,47,113–116] due to conversion of natural vegetation to grasslands on the Amazonian agricultural frontier.

Results from other tropical catchments studies that show a decrease in dry season streamflow as a consequence of forest conversion [51,117] cannot be confirmed in our study in the Cerrado biome. From the wet to the dry season our results showed a greater decrease in streamflow in the cerrado catchment than in the pasture catchment, while the E_T_ behaved otherwise with lower decrease in the cerrado catchment. We suggest that this is related to the higher root zone storage capacity of the cerrado vegetation. The deep roots of the cerrado vegetation influence the water balance and appear to be important in proving water for vegetation during the dry season [118]. Indeed, the cerrado vegetation is highly adapted to a long dry season and deeply weathered soils [27], which is a particular situation that demands more detailed hydrological research in this region. The replacement of the cerrado vegetation with exotic grasses seems to increase the deep seepage and reduce E_T_, which in turn will increase the streamflow, especially during the dry season.

## Conclusions

We investigated the hydrological responses of two headwater micro-catchments with contrasting land use (cerrado vs. pasture) in the Brazilian Cerrado using field data collected between 2012 and 2014. From our study, we conclude that the conversion of the undisturbed cerrado to pasture caused:

1. Significant soil hydro-physical degradation as indicated by higher bulk density and reduced soil porosity in the pasture catchment in comparison to the cerrado catchment;
2. An increase in streamflow as shown by the significantly greater daily and annual streamflow values in the pasture catchment. Furthermore, we conclude that cerrado conversion to pasture reduced the evapotranspiration.

While our study contributes to understanding of the soil degradation and hydrological processes in this region, we suggest long-term measurements including quantifying changes in groundwater storage in order to better clarify the mechanisms causing the observed behavior in our data.
