# Correction: Effects of conversion of native cerrado vegetation to pasture on soil hydro-physical properties, evapotranspiration and streamflow on the Amazonian agricultural frontier

**DOI:** 10.1371/journal.pone.0236236

**Published:** 2020-07-17

**Authors:** Rodolfo L. B. Nóbrega, Alphonce C. Guzha, Gilmar N. Torres, Kristof Kovacs, Gabriele Lamparter, Ricardo S. S. Amorim, Eduardo Couto, Gerhard Gerold

Fig 5 is incorrect because it exhibits monthly rainfall maxima and not the monthly total. The analyses, results and data provided with the publication remain correct. The authors apologize for this error in the original publication and have provided a corrected version of Fig 5 here.

**Fig 5:**
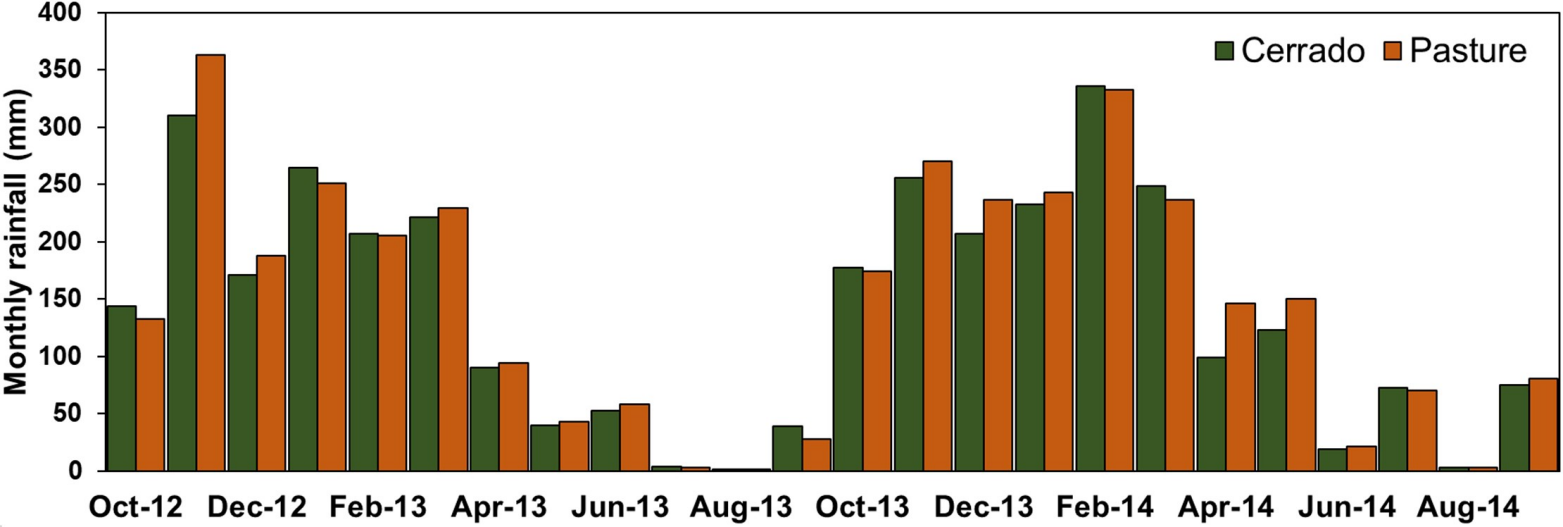
Monthly rainfall per catchment.
